# Molecular insights into novel environmental strains of *Klebsiella quasipneumoniae* harboring different antimicrobial-resistance genes

**DOI:** 10.3389/fpubh.2022.1068888

**Published:** 2023-01-12

**Authors:** Hisham N. Altayb, Salman Hosawi, Othman Baothman, Imran Kazmi, Kamel Chaieb, Isam M. Abu Zeid, Hana S. Elbadawi, Bruno Silvester Lopes, Ehssan Moglad

**Affiliations:** ^1^Department of Biochemistry, Faculty of Science, King Abdulaziz University, Jeddah, Saudi Arabia; ^2^Center of Artificial Intelligence in Precision Medicines, King Abdulaziz University, Jeddah, Saudi Arabia; ^3^Department of Biological Sciences, Faculty of Science, King Abdulaziz University, Jeddah, Saudi Arabia; ^4^Microbiology and Parasitology Department, Soba University Hospital, University of Khartoum, Khartoum, Sudan; ^5^School of Health and Life Sciences, Teesside University, Middlesbrough, United Kingdom; ^6^National Horizons Centre, Teesside University, Darlington, United Kingdom; ^7^Department of Pharmaceutics, College of Pharmacy, Prince Sattam bin Abdulaziz University, Al-Kharj, Saudi Arabia

**Keywords:** *K. quasipneumoniae* subsp. *similipneumoniae*, *K. quasipneumoniae* subsp. *quasipneumoniae*, misidentification, whole-genome sequencing, antimicrobial resistance

## Abstract

**Introduction:**

The emergence of bacterial pathogens in environmental hosts represents a major risk to public health. This study aimed at characterizing seven novel environmental strains of *K. quasipneumoniae* using a genomic approach which was misidentified by phenotypic methods in a previous batch of 27 species thought to be *K. pneumoniae*.

**Methods:**

Whole-genome sequencing was performed using the Illumina platform, and the generated raw reads were *de novo* assembled. Comparative genomic, resistome, virulome, mobilome, and phylogeny were then investigated using dierent bioinformatics tools.

**Results:**

Six strains were identified as *K. quasipneumoniae* subsp *similipneumoniae* and one as *K. quasipneumoniae* subsp. *quasipneumoniae*. All isolates were resistant to ampicillin, cephalexin, and amoxicillin-clavulanic acid and harbored the *fosA*, *bla*_OKP_ types, *oqxB*, and *oqxA* genes. One isolate additionally harbored a gene cassettes consisting of *bla*_SHV−1_, *bla*_OXA−1_, *aac(6*′*)-Ib-cr, catB* genes. The aminoglycoside-modifying enzyme gene *aph(3”)-Ia* was bracketed by two insertion elements. Plasmid analyses showed that IncFIB_K_ was the most prevalent plasmid, circulating in six isolates, while one isolate exhibited seven different plasmids. The isolates have virulence genes responsible for capsule formation, lipopolysaccharide, iron uptake aerobactin (*iutA*), salmochelins (*iroE, iroN*), enterobactin siderophore, adherence, and biofilm formation (*mrkA, mrkB, mrkC, mrkD, mrkF*, and *mrkH*).

**Conclusion:**

Our study highlights the ecology and transmission of *K. quasipneumoniae* (which have the ability to disseminate to other environmental sources including animals) outside the clinical setting and the contribution of water, vegetables, and table surfaces as potential reservoirs of farm-to-fork transmission of disease via local markets in Khartoum, Sudan.

## Introduction

*Klebsiella pneumoniae* represents an ongoing and growing challenge for humans and is classified as a critical priority pathogen listed by the WHO. Hypervirulent community-acquired invasive strains of *Klebsiella* represent a major problem in developing countries with the growing threat of convergence of multidrug-resistant (MDR) and hypervirulent phenotypes (1). *K. pneumoniae* is not only capable of causing serious clinical infections but can acquire significant levels of antibiotic resistance genes leading to failure in the treatment of patients with urinary tract and respiratory tract infections, liver abscess, endophthalmitis, and meningitis (2). *K. pneumoniae* is a familiar MDR nosocomial pathogen and has the capacity to adapt to various environmental and clinical settings (3). Recent taxonomic classification has delineated at least seven phylogroups of *K. pneumoniae* (Kp1–Kp7) which contribute to the *K. pneumoniae* species complex (KpSC). The KpSC consists of five species, these include *K. pneumoniae sensu stricto* (Kp1), *K. quasipneumoniae* [subsp. *quasipneumoniae* (Kp2) and subsp. *similipneumoniae* (Kp4)], *K. variicola* [subsp. *variicola* (Kp3) and subsp. *tropica* (Kp5)] and *K. quasivariicola* (Kp6, which is not formally defined yet), and *K. africana* (Kp7) (4–6). Originally, *K. quasipneumoniae* was found to be associated with the environment. However, there are now increasing reports of it being a human pathogen associated which underlying hospital-acquired infections (7, 8). Identification of *K. quasipneumoniae* is challenging when using conventional laboratory methods due to the similarity in their biochemical tests with other *Klebsiella* spp. leading to misidentification and false reporting (5).

Multidrug-resistant (MDR) hypervirulent strains of *K. quasipneumoniae* subsp. *quasipneumoniae* are becoming an issue for public health worldwide. Emerging antimicrobial resistance genes (ARGs) conferring resistance to last-resort antibiotics including cephalosporins, carbapenems, and tigecycline, have been reported in clinical and environmental isolates. Such genes include *bla*_CTX−M−2_, *bla*_DHA−1_, *bla*_NDM_, *bla*_IMP_, *bla*_KHM−1_, *bla*_OXA−48_, *bla*_OXA−162_, *bla*_OXA−10_, *bla*_KPC_, *tet*(A), and *tet(K)* genes (9–12). Environmental settings, especially water surfaces, soil, animal waste, and sewage treatment plants, are implicated in promoting the distribution of ARGs and as a result act as a reservoir of multidrug-resistant (MDR) *K. quasipneumoniae* (13).

Furthermore, *K. quasipneumoniae* has the ability to uptake ARGs and plasmids from other Enterobacteria, including plasmids belonging to different incompatibility groups such as IncU/IncX5 which harbors *bla*_KPC_, IncHI2 which harbors *mcr-9*, and IncFII/IncFIB which harbors *mcr-8.2* (7).

The emergence of MDR pathogens in the environment, particularly from a one-health perspective is hence becoming a global threat to public health (14). *K. quasipneumoniae* has been identified as an opportunistic pathogen capable of harboring different ARGs that render clinically important antibiotics ineffective. Hence, this study aimed at characterizing the genomes of seven *K. quasipneumoniae* isolated from environmental sources such as water, vegetables, and table surfaces from the local market in Khartoum Bahre, Sudan.

## Materials and methods

### Isolate selection

A total of 27 bacterial isolates were collected from clinical (*n* = 10) and environmental samples (*n* = 17) between March and July 2021, the isolates were initially identified as *K. pneumoniae* using standard biochemical tests (15). Among the *K. pneumoniae* (*n* = 27) 7 were reidentified as *K. quasipneumoniae* using genomic tools. More details on the rest of *K. pneumoniae* (*n* = 20) isolates, sample collection, and processing have been published elsewhere (16). The environmental samples were obtained via swabs collected from vegetables and table surfaces in the vegetable market in the Khartoum locality. In addition, water samples were collected from water stations in the Khartoum locality. The samples were collected and processed according to published protocols (17, 18). Chromogenic UTI agar media (bioMérieux, France) and MacConkey agar (HiMedia, India) were used for the differentiation of the isolates according to their phenotypic characteristics. Standard biochemical tests were used for the identification of the isolates (15).

### Antimicrobial sensitivity testing and minimum inhibitory concentration

Disk-diffusion method was used for the determination of antimicrobial susceptibility testing. The antibiotics tested were, amoxicillin-clavulanate (30 μg), cefuroxime (30 μg), ceftriaxone (30 μg), ceftazidime (30 μg), cephalexin (30 μg), meropenem (10 μg), imipenem (10 μg), amikacin (30 μg), gentamicin (10 μg), ciprofloxacin (5 μg), trimethoprim-sulfamethoxazole (25 μg), and chloramphenicol (10 μg). Minimum inhibitory concentration (MIC) was performed for ampicillin, chloramphenicol and ciprofloxacin using the broth dilution method in a serial dilution ranging from 0.5 to 256 μg/ml (19). *K. pneumoniae* ATCC 700603 and *E. coli* ATCC 25922 were used as quality controls and the results were interpreted according to the CLSI guidelines (20).

### Whole-genome sequencing and molecular analysis

DNA was extracted from overnight bacterial growth of pure colonies on Nutrient Agar (HiMedia, India), by the quinidine chloride protocol as described by Sabeel et al. (21). The DNA quality was checked by gel electrophoresis where clear crisp bands indicate DNA of high quality and on the contrary, a smear indicates DNA degradation or low quality. It was also quantitatively checked using nanodrop and Qubit (Thermo Scientific, USA). The extracted DNA was subjected to next-generation sequencing with 100X coverage. Paired-end (2 × 150 bp) whole-genome sequencing was achieved using Illumina HiSeq 2500 platform (Illumina, USA), by Novogene Company (China). PATRIC server assembly was used to obtain *de-novo* assemblies. Identification of species and strains was performed by MLST 2.0 and PubMLST (22). The assignment of the new sequence types and subspecies identification were achieved by the Pasteur MLST database curators. Genomes were annotated by PATRIC server and NCBI Prokaryotic Genome Annotation Pipeline (PGAP) (23). The Pathogenwatch platform was used for capsule (K) and O serotype identification (24).

The resistome profile was analyzed by Resistance Gene Identifier (RGI) and ResFinder (25). Mobile genetic elements, insertion sequences and plasmids were identified by Mobile Element Finder (26), IS Finder, and Plasmid Finder 2.1, respectively. Geneious Prime was used for the visualization of resistant genes and transposon cassettes. Virulence factors were investigated using VirulenceFinder 2.0 and the Virulence Factor Database (VFDB) (27) where genomes of *K. quasipneumoniae* subsp. *quasipneumoniae* (18A069 and MGH96) and *K. quasipneumoniae* subsp. *similipneumoniae* (HKUOPA4, HKUOPL4, and ATCC700603) were used as reference.

### Core-genome and average nucleotide identity analysis

Core-genome multi-locus sequence typing (cgMLST) classification and core genome MLST profile comparison were achieved using Pathogenwatch, which includes tools that infer *Klebsiella* lineage codes based on references from the Pasteur/PubMLST resource.

Average nucleotide identity analysis was conducted using fastANI v. 1.33 (28). For ANI analysis of our isolates, five reference genomes were used for comparison, which includes *K. quasipneumoniae* subsp. *quasipneumoniae* (18A069 and MGH96) and *K. quasipneumoniae* subsp. similipneumoniae (HKUOPA4, HKUOPL4, and ATCC700603). Heat map and SNP matrix were built by FastANI_heatmap using R code for building a heat map and histogram with the output of FastANI. ANI with more than 95% identity was considered suitable to identify species and ≥ 98% to identify subspecies (29–31).

### Comparative genomics and phylogenomics analysis

A comparative genomics study was performed using the Gview tools (32) and PATRIC Proteome Comparison tool (33). The phylogenetic tree was generated by PATRIC phylogenetic tree builder, using the assembled contigs as input. In PATRIC the phylogenetic tree was created by using Codon Tree approach, which utilized PATRIC global protein families (PGFams) as homology groups. A group of PGFams were discovered among these chosen genomes using the Codon Tree analysis, and the aligned proteins and coding DNA from single-copy genes were used for RAxML analysis (34). The tree was built for the seven isolates of *K. quasipneumoniae* and the most similar genomes of *K. quasipneumoniae* (*n* = 27) from NCBI database. Figtree (35) was used for the modification and visualization of the generated tree.

### Nucleotide sequence accession numbers

The Bioproject for isolates is PRJNA767482, and the complete chromosomal sequences were submitted to GenBank under accessions JAJOZL000000000, JAJOZM000000000, JAJOZN000000000, JAJOZO000000000, JAJHNR000000000, JAJOZP000000000, and JAJONG000000000 for isolates 3KE, 4KE, 5KE, 6KE, 8KE, 10KE, and 14KE respectively.

## Results

### Isolates identification

From a batch of 27 *Klebsiella* spp. identified phenotypically as *K. pneumoniae*, 7 (26%) were primarily misidentified as *K. pneumoniae* which after WGS were reidentified as *K. quasipneumoniae* by MLST 2.0, and PubMLST databases.

### Antimicrobial susceptibility testing and MIC

All isolates were resistant to ampicillin, amoxicillin-clavulanic acid and cephalexin, while they were sensitive to meropenem, imipenem, amikacin, gentamicin, ciprofloxacin, ceftazidime, ceftriaxone, cefuroxime, chloramphenicol, and trimethoprim-sulfamethoxazole (Table 1).

**Table 1:** The minimum inhibitory concentration (μg/ml) of antibiotics against *K. quasipneumoniae* isolates.

**ID**	**Location**	**Sample source**	**AMP**	**CIP**	**C**
3KE	Market in Bahre	Water	256	2	2
4KE	Market in Bahre	Water	128	0.5	2
5KE	Market in Bahre	Vegetable	256	0.5	2
6KE	Market in Bahre	Vegetable	128	2	2
8KE	Market in Bahre	Water	256	2	4
10KE	Market in Khartoum	Vegetable	256	2	2
14KE	Market in Khartoum	Table surface	128	4	4
ATCC 700603	–	–	128	0.5	32

AMP, ampicillin; CIP, ciprofloxacin; C, chloramphenicol.

### Genomic features and sequence types of the isolate

All assembled reads had coverage in a range of 171–198, an average genomic length of 5,320,537 bp, a number of contigs in a range of 213–386, GC content of 57%, and an average number of coding sequences (CDS) of 5,322 (Table 2).

**Table 2:** Genomic features of different strains of *K. quasipneumoniae*.

**ID**	**PubMLST species**	**Identity**	**BIGSdb-pasteur subsp assignment**	**BIGSdb-ID**	**ST type**	**Capsule (K_locus)**	**Predicted O type**	**Genome size bp**	**Contigs**	**GC**	**CDS**	**tRNA**	**rRNA**	**Coverage**	**N50**
3KE	*K. quasipneumoniae*	100%	*K. quasipneumoniae* subsp. *similipneumoniae*	18,580	5,926	KL114	O3/O3a	5,338,925	227	57.84	5,198	39	6	188	50,455
4KE	*K. quasipneumoniae*	100%	*K. quasipneumoniae* subsp. *quasipneumoniae*	18,615	4,768	KL114	O3/O3a	5,466,338	309	57.82	5381	42	4	171	50,431
5KE	*K. quasipneumoniae*	100%	*K. quasipneumoniae* subsp. *similipneumoniae*	18,616	2,019	KL66	O12	5,256,549	233	57.96	5100	39	5	198	52,257
6KE	*K. quasipneumoniae*	98%	*K. quasipneumoniae* subsp. *similipneumoniae*	18,617	5,944	KL66	O12	5,535,420	386	57.02	5113	41	5	190	51,677
8KE	*K. quasipneumoniae*	100%	*K. quasipneumoniae* subsp. *similipneumoniae*	1,505	5,922	KL81	unknown (OL101)	5,258,392	263	57.88	5137	38	4	190	50,455
10KE	*K. quasipneumoniae*	98%	*K. quasipneumoniae* subsp. *similipneumoniae*	18,618	5,945	KL146	O12	5,109,309	213	58.00	4927	38	6	191	53,634
14KE	*K. quasipneumoniae*	100%	*K. quasipneumoniae* subsp. *similipneumoniae*	18,581	5,927	KL166	O3/O3a	5,362,480	393	57.74	5348	43	5	174	39,683

When queried with the rMLST database sited at PubMLST, five isolates showed 100% identity, and two showed 98% identity to *K. quasipneumoniae* (https://pubmlst.org/bigsdb?db=pubmlst_rmlst_seqdef_kiosk, last accessed, on 21st September 2022). The isolates were subsequently identified as *K. quasipneumoniae* subsp. *similipneumoniae* (*n* = 6) and *K. quasipneumoniae* subsp. *quasipneumoniae* (*n* = 1). Six isolates were identified at the subspecies level and assigned the novel sequence types and IDs by the Institut Pasteur team (Table 2). The isolate 5KE was not defined as a novel ST due to partial sequence alignment of *tonB* gene alleles. Isolate 4KE was identified with unknown sublineage, clonal group and core genome sequence type (cgST), while isolates 5KE, 6KE, and 8KE were identified with known clonal group, and cgST as shown in Supplementary material 1 and Supplementary Table 1.

Prediction of the capsule (K) and O serotypes revealed that isolates 3KE and 4KE possessed KL114 capsule-type whereas isolates 5KE, 6KE, and 14KE harbored the KL66 K-loci. The KL81 and KL146 capsule (K) types were present in 8KE and 10KE respectively. Isolates 3KE, 4KE, and 14KE belonged to O3/O3a serotype and isolates 5KE, 6KE, and 19KE had the O12 serotype. In isolate 8KE the serotype was unknown (Table 2).

**Figure 1:**
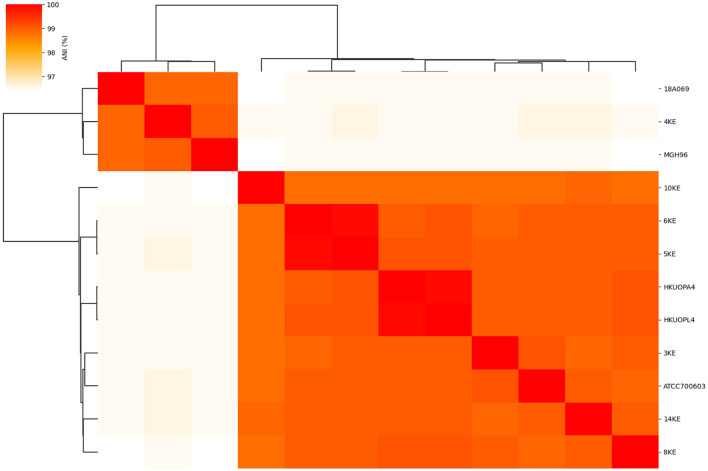
Heat map of the average nucleotide identity (ANI) and phylogenetic tree of subspecies of *K. quasipneumoniae* strains.

ANI analysis was performed on the whole genome sequences for species and subspecies identification; the 95% identity criteria were considered for species identification, and 98% for subspecies identification. The *K. quasipneumoniae* subsp. *quasipneumoniae* (4KE) strain showed an average of 96.5% ANI compared to other *K. quasipneumoniae* subsp. *similipneumoniae* strains, while the 4KE showed 99% identity with reference strains of the same subspecies *K*. *quasipneumoniae* subsp. *quasipneumoniae* reference strains (18A069 and MGH96). At the same time, the comparison of *K. quasipneumoniae* subsp. *similipneumoniae* strains (3KE, 5KE, 6KE, 8KE, 10KE, and 14KE) showed high ANI (ranging from 98.84 to 99.96%) similarity with *K. quasipneumoniae* subsp. *Similipneumoniae* reference strains (HKUOPA4, HKUOPL4 and ATCC700603). 5KE and 6KE were the closest strains showing 99.96% identity, while 10KE showed the lowest (with an average of 98.87%) identity among subspecies of *K. quasipneumoniae* subsp. *similipneumoniae* (Table 3, Figure 1). Alleles differences generated by Pathogenwatch are described using distance matrices as shown in Supplementary Table 2.

**Table 3:** ANIs comparison among subspecies of *K. quasipneumoniae* subsp. *quasipneumoniae* and *K. quasipneumoniae* subsp. *similipneumoniae*.

**Strain**	**18A069**	**4KE**	**MGH96**	**10KE**	**6KE**	**5KE**	**HKUOPA4**	**HKUOPL4**	**3KE**	**ATCC700603**	**14KE**	**8KE**
18A069	100	98.92	98.99	96.49	96.52	96.53	96.56	96.53	96.55	96.53	96.52	96.45
4KE	98.925	100	99.00	96.54	96.57	96.60	96.55	96.59	96.59	96.61	96.64	96.58
MGH96	98.99	99.00	100	96.46	96.50	96.53	96.55	96.54	96.54	96.56	96.54	96.46
10KE	96.49	96.54	96.46	100	98.86	98.84	98.87	98.89	98.88	98.86	98.93	98.84
6KE	96.52	96.57	96.50	98.86	100	99.96	99.09	99.10	98.99	99.04	99.06	99.07
5KE	96.53	96.60	96.53	98.84	99.96	100	99.11	99.13	99.00	99.03	99.06	99.08
HKUOPA4	96.56	96.55	96.55	98.87	99.09	99.11	100	99.99	99.03	99.05	99.05	99.11
HKUOPL4	96.53	96.59	96.54	98.89	99.10	99.13	99.99	100	99.03	99.04	99.06	99.14
3KE	96.55	96.59	96.54	98.88	98.99	99.00	99.03	99.03	100	99.12	98.92	99.03
ATCC700603	96.53	96.61	96.56	98.86	99.04	99.03	99.05	99.04	99.12	100	99.05	98.92
14KE	96.52	96.64	96.54	98.93	99.06	99.06	99.05	99.06	98.92	99.05	100	99.01
8KE	96.45	96.58	96.46	98.84	99.07	99.08	99.11	99.14	99.03	98.92	99.01	100

### Predicted antimicrobial resistance mechanism, virulence genes, and plasmids

**Figure 2:**
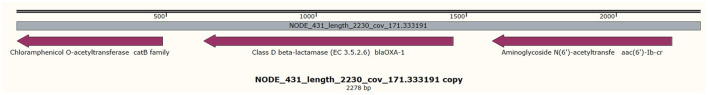
Clustering of bla_OXA−1_, aac(6')-Ib-cr, and catB genes in contig 431 in isolate 6KE, the gray middle line indicates the length, and coverage of the contig, and the purple arrows indicate the reported genes and their orientations.

**Figure 3:**
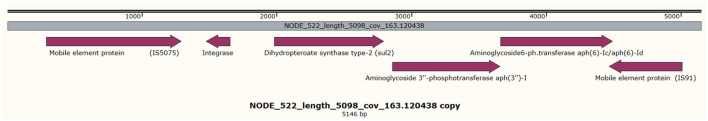
Showing the presence of aph(3′)-Ib, sul2 genes harbored in isolate 6KE and is found in brackets of two mobile elements. The gray middle line indicates the length, and coverage of the contig, and the purple arrows indicate the reported genes and their orientations.

All the isolates harbored the fosfomycin resistance gene (*fosA)*, the chromosomally expressed beta-lactamases (*bla*_OKP_ types), and olaquindox/quinolone AB (*oqxAB*) efflux pump genes. The *ompK36* and *ompK37* genes involved in reduced permeability to beta-lactams were present in all the isolates except 5KE. The *fosA6* gene was present only in isolate 4KE, and the *fosA7* gene was present only in isolate 3KE. Isolate 6KE harbored a cluster of *bla*_SHV−1_, *bla*_OXA−1_, *aac(6*′*)-Ib-cr*, and *catB* (Table 4, Figure 2). The aminoglycoside-modifying enzyme gene [*aph(3”)-Ia*] was harbored in isolate 6KE and was bracketed by two transposable insertion elements IS*5075* at 5′ and IS*91* transposase at the 3′ ends (Figure 3).

**Table 4:** Antibiotic resistance genes and mobile genetic elements present in *K. quasipneumoniae* isolates.

**ID**	**ARGs**	**Virulence gene**	**Insertion elements/Integrase**
3KE	*fosA, bla_*OKP*−*B*−10_, OqxB, OqxA, fosA7, ompK36, ompK37*	*iutA*	–
4KE	*bla_*OKP*−*A*−_3, OqxB, OqxA, fosA6, fosA, ompK36, ompK37*	*iutA*	IS*Sty2*, IS*Kpn26*, IS*Kpn21*, IS*Kpn43*, IS*Ec52*
5KE	*fosA, OqxB, OqxA, bla_*OKP*−*B*−2_*	*iutA, traT*	IS*Kpn43*, IS*421*, IS*26*
6KE	*fosA, OqxB, OqxA, bla_*OKP*−*B*−2_, ompK36, ompK37, bla_*SHV*−1_, bla_*OXA*−1_, aph(3”)-Ib, AAC(6')-Ib-cr, CatB*	*iutA, traT*	IS*Kpn43*, IS*421*, IS*26*, integrase IntI1
8KE	*fosA, bla_*OKP*−*B*−10_, OqxB, OqxA*	–	–
10KE	*fosA, bla_*OKP*−*B*−14_, OqxB, OqxA, ompK36, ompK37*	*iutA*	IS*Ecl10*
14KE	*fosA, bla_*OKP*−*B*−14_, OqxB, OqxA, ompK36, ompK37*	*iutA*	IS*Kpn28*, IS*26*, IS*Kpn21*

The most common efflux pumps identified in the isolates were *KpnG, LptD, CRP, eptB, ArnT, KpnF*. Gene point mutations associated with drug resistance were also investigated and the acrR: Y114F, V165I, and EF-Tu: R234F point mutations were identified commonly in the isolates (Supplementary material 1, Supplementary Table 3).

Upon investigation of virulence factors, the type 3 fimbriae adherence-related genes were more common in the isolates. These were the major fimbrial subunit (*mrkA*), chaperone (*mrkB*), usher (*mrkC*), adhesins (*mrkD, mrkH*), and minor fimbrial subunit (*mrkF*). All type I fimbriae adherence-related genes (*fim* A-H) were identified in isolates 3KE, 6KE, 10KE, 14KE and the reference strains, while isolates 4KE lacked *fimA*, and *fimF* genes. Isolate 5KE lacked the *fimB*, while isolate 8KE possessed only *fimD*. The iron uptake aerobactin (*iutA)*, salmochelins (*iroE, iroN*), and enterobactin siderophores (*ent* A-F, *feb* A-G), *entS*, and *fes* were identified in all the isolates, with notable exceptions: the *iutA* gene which was absent in 8KE, the *entD* gene was absent in 6KE and *fes* gene was absent in 4KE. Isolate 8KE was characterized by the presence of 16 types of secretion systems **[**T6SS-I (15) and (T6SS-II)], while 4KE was characterized by the presence of 14 types of T6SS-III. Six lipopolysaccharides biosynthetic (*rfb*) loci were identified in isolates 3KE, 4KE, 14KE, and the reference ATCC 35657, while only one locus was identified in the rest of the isolates except the reference MGH96 which is lacked all the lipopolysaccharide genes (Supplementary material 1, Supplementary Table 4).

Plasmid prediction showed that IncFIB_K_ was the most prevalent plasmid, circulating in six isolates; one isolate carried two plasmids IncFIB_K_ Col440I, while other strains carried five plasmids ColpVC, IncN, IncFIB_K_, IncFII_pKPX1_, and IncR. Isolate 4KE contained 7 plasmids: Col_pHAD28_, Col_pHAD28_, ColpVC, IncFIB_K_, IncFII_pKPX1_, IncN, and IncR (Table 5).

**Table 5:** Predicted plasmids in *K. quasipneumoniae*.

**Isolate**	**Plasmid**	**Identity**	**Query/template length**	**Contig**	**Position in contig**	**Accession number**
3KE	IncFIB_K_(*p*CAV1099*-*114)	95.36	560/560	129	3355.3914	CP011596
4KE	Col_pHAD28_	96.43	84/131	57	3..86	KU674895
	Col_pHAD28_	95.92	98/131	316	3..100	KU674895
	ColpVC	97.93	193/193	60	1289..1481	JX133088
	IncFIB_K_	98.75	560/560	661	3345..3904	JN233704
	IncFII_pKPX1_	97.23	577/577	286	1213..1789	AP012055
	IncN	99.42	514/514	68	256..769	AY046276
	IncR	99.47	188/251	260	17208..17395	DQ449578
5KE	IncFIB_K_	98.93	560/560	308	1938..2497	JN233704
6KE	IncFIB_K_	98.93	560/560	316	1938..2497	JN233704
8KE	IncFIB_K_	98.93	560/560	87	3314..3873	JN233704
10KE	NIL					
14KE	Col440I	96.2	79/114	66	827..905	CP023920
	IncFIB_K_	98.57	560/560	452	3353..3912	JN233704

Antimicrobial susceptibility testing was interpreted according to CLSI guidelines (20).

### Comparative genomics

**Figure 4:**
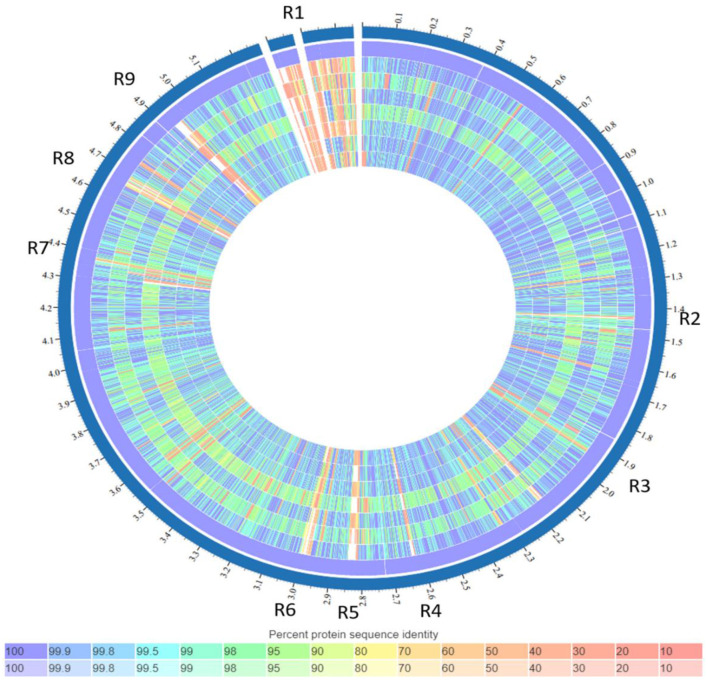
List of tracks, from outside to inside: *K. quasipneumoniae* strain ATCC 700603, isolate 3KE, isolate 4KE, isolate 5KE, isolate 6KE, isolate 10KE, isolate 14KE, isolate 8KE. Regions containing unique genes or features (named R1–R9). Colors indicate the percent protein identity of sequences to the reference, blue indicates high similarity (100%) while red indicates low similarity (0%).

**Figure 5:**
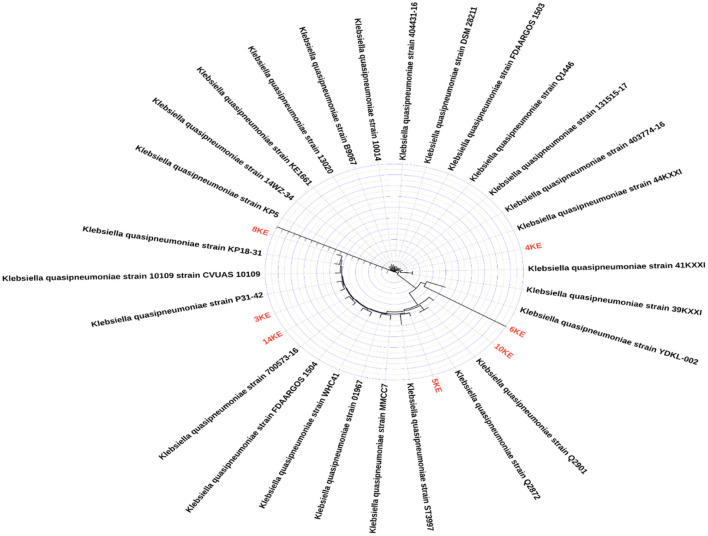
Phylogenetic tree of our isolates colored red and other *K. quasipneumoniae* isolates from Genbank database (n = 27).

The comparison of the genomes of *K. quasipneumoniae* isolates (3KE, 4KE, 5KE, 6KE, 8KE, 10KE, and 14KE) with *K. quasipneumoniae* strain ATCC 700603 revealed 9 unique regions among our isolates (Figure 4). These regions contain different types of unique proteins; the R1 region contains mobile elements (plasmids, AMR genes, and transposases), proteins associated with phage integration, anti-restriction protein klcA, and hypothetical proteins. Isolate 4KE contains, exclusively in region 1, a unique set of IncI1 plasmid conjugative transfer proteins and they include: IncI1 plasmid conjugative transfer protein TraU, IncI1 plasmid conjugative transfer protein TraW, IncI1 plasmid conjugative transfer protein TraX, IncI1 plasmid conjugative transfer integral membrane protein TraY, and IncI1 plasmid conjugative transfer protein TraQ. Region 2 contains transposases and phage-associated proteins, while region 3 contains proteins that regulate the length and adhesion of type 1 fimbriae and contains several genes involved in capsule production, fimbrial elements, and putative transcriptional regulatory proteins. Isolates 4KE, 5KE, 8KE, and 10KE are harbored in regions 4, 5, and 9 phages and phages-associated proteins in addition to hypothetical proteins (Supplementary material 2). Phylogenetic analysis revealed that isolates 3KE, 5KE, 8KE, 10KE, and 14KE were clustered at the same branch, indicating their relatedness, in contrast with 6KE and 4KE, which formed two separate branches (Figure 5).

Resistome comparison among our isolates and reference strains, showed the unique presence of antimicrobial resistance genes *bla*_*SHV*−1_, *bla*_*OXA*−1_*, aph(3”)-Ib, AAC(6*′*)-Ib-cr*, and *CatB* in 6KE. The *fosA, bla*_*OKP*_*, OqxB, OqxA*, and *fosA7* were commonly identified in all isolates including references (Supplementary material 1, Supplementary Table 1).

The virulome comparison revealed the presence of type I secretory system (T6SS-I) associated genes (*clpV/tssH, dotU/tssL, hcp/tssD, icmF/tssM, ompA, sciN/tssJ, tssF, tssG, vasE/tssK, vgrG/tssI, vipA/tssB*, and vipB*/tssC*) only in isolate 8KE and three reference strains (ATCC35657, MGH96, and 18A069), while the *clpV* gene was found to be dominant in all isolates. Type III secretory system (T6SS-III) virulence genes (*dotU, icmF, impA, impF, impG, impH, impJ, ompA, sciN*, and *vgrG*) have been identified in isolate 4KE and three reference strains (ATCC35657, MGH96, and 18A069). Five LPS rfb loci have been identified in isolates 3KE and 4KE and two reference strains (ATCC35657 and 18A069). Fimbrial adherence determinants of Salmonella species (*stcB*) have been identified in 3KE and 10KE genes (Supplementary material 1, Supplementary Table 4).

Different plasmids were identified in our isolates: seven plasmids (ColpHAD28, ColpHAD28, ColpVC, IncFIB_K_, IncFII_pKPX1_, IncN, and IncR) were identified in 4KE isolate; IncFIB_K_ plasmid was identified in 3KE, 5KE, 6KE, 8KE, and 14KE, while there was no plasmid identified in reference isolates.

## Discussion

The emergence of bacterial pathogens in environmental niches represents a continuous risk to human health (36). *K. quasipneumoniae* is a newly identified bacterial species discovered in 2014, which has not been fully understood until now (7). Due to the large similarity between *K. variicola, K. pneumoniae*, and *K. quasipneumoniae* isolates, there is difficulty in their identification and classification using routine biochemical tests (5, 37, 38). Additionally, identification based on public databases represents a source of misidentification due to the wrong submission to these databases (39). This may lead to misclassification even by using WGS. In this study, 26% (7/27) of *K. quasipneumoniae* species were misidentified as *K. pneumoniae* as a part of a previous study intended to study the genomics of *K. pneumoniae* and *E. coli* (16). This finding is consistent with Long et al. who showed that 30% of *K. pneumoniae* are misidentified by conventional biochemical tests (40).

cgMLST, core-genome SNP analysis, and ANI schemes demonstrated their ability to subtype species with the same MLST denominations (28, 31, 41). ANI with more than 95% identity was considered suitable to identify species and ≥98% to identify subspecies (29–31). In this study, the comparison of *K. quasipneumoniae* subsp. *quasipneumoniae* and *K. quasipneumoniae* subsp. *similipneumoniae* showed an average ANI of 96.5%, while the comparison of the same subspecies showed ANI ranging from 98.84 to 99.96%, supporting their subspecies relativeness. These findings are in line with Nicolás et al. (37) who found the same difference (96.52%) between subspecies of *K. quasipneumoniae* subsp. *quasipneumoniae* and *K. quasipneumoniae* subsp. *Similipneumoniae;* they also found 99% similarity among the same subspecies.

Our results showed that one strain (6KE) possessed a variety of genes such as *aph(3”)-Ib, aac(6*′*)-Ib-cr*, and c*atB*, which conferred resistance to aminoglycosides and chloramphenicol, but it was phenotypically sensitive to these antibiotics. Although gene sequences were intact, the activity was not observed, which may be attributed to their low expression level (42). Isolates expressed c*atB* and sensitive to chloramphenicol were previously reported (43), which was attributed to the decreased levels of acetyl coenzyme A in the isolates.

Furthermore, the presence of plasmids, insertion sequences (IS*Kpn43*, IS*421*, and IS*26*), and integron integrase (*intI1*) can aid in the mobility of AMR genes which could increase the risk of transmission and dissemination of resistance in other susceptible strains (42, 44, 45).

Although *Klebsiella* species are intrinsically resistant to ampicillin (46), in this study we noted a high resistance rate to ampicillin (MIC ≥ 128 μg/ml). This could be attributed to the presence of different variants of class A beta-lactamases (*bla*_OKP_) that are associated with intrinsic ampicillin resistance in *Klebsiella* species (47, 48). This finding is higher than what was reported recently in Saudi Arabia for ampicillin-resistant *K. quasipneumoniae* (MIC = 32 μg/ml) (38). In this study, the isolates were resistant to cephalexin and ampicillin, while being susceptible to other beta-lactam antibiotics (meropenem, imipenem, ceftazidime, ceftriaxone, and cefuroxime). Although *Klebsiella* species have no intrinsic resistance to cephalosporins, the chromosomally expressed *bla*_OKP_ beta-lactamases can cause a low level of resistance to cephalexin (47).

Plasmid screening showed that IncFIB_K_ was the most prevalent plasmid, circulating in six isolates except 10KE. The IncFIB_K_ plasmid is known to be a vehicle for the transmission of AMR genes in *Enterobacterales* (49). This plasmid is more common in environmental isolates of *K. quasipneumoniae* and has also been documented commonly in clinical isolates of *K. pneumoniae* (50), which supports our finding.

Several key features are common in our isolates when compared to *K. quasipneumoniae* strain ATCC 700603, such as putative transcriptional regulatory protein flanking Lysine 2,3-aminomutase (EC 5.4.3.2) which is involved in Lysine degradation (51). This was uniquely detected in all isolates. Genes encoding methyl-directed repair DNA adenine methylase, and twitching motility protein (*PilT*) which is associated with the surface-associated bacterial movement, were observed as mutations in *PilT* and *pilU* in *Pseudomonas aeruginosa* and are shown to be defective twitching motility (52). Antirestriction protein klcA was reported in all isolates and is located near incF plasmid proteins; the presence of anti-restriction protein klcA_HS_ has been reported to increase plasmid transformation by 3–6 folds thereby increasing competence (53).

Genes associated with drug resistance and virulence mechanism are used for differentiating the commensals from pathogenic bacteria (54). The virulence factors investigated in our isolates showed aerobactin siderophore receptor gene (*iutA*) and salmochelin (*iroE, iroN*) genes commonly reported in the isolates, and are considered as clear markers of hypervirulent strains of *K. pneumoniae* (55). These genes were reported in *K. pneumoniae* strains isolated recently from the same location in Sudan (Khartoum locality) (56). Additionally, the type 3 fimbrial proteins (mrkA, mrkB, mrkC, mrkD, mrkF, and mrkH) were detected in all the isolates, which could increase the chance of biofilm production (57); isolates producing biofilm are associated with hospital-acquired infections and chronic infections (58). In addition to the presence of type 3 fimbrial (*mrkD*) gene, the isolates possessed type III secretory system (T6SS-III). Isolates positive for *mrkD* and T6SS-III fimbriae can establish biofilm formation in harsh environments (59).

The key virulence factors of pathogenic *Klebsiella* spp. are two cell surface-associated glycoproteins called capsular polysaccharides (CPS) and lipopolysaccharides (LPS) (60). In this study, the analysis of the capsule structure of the isolates revealed that isolates 3KE and 4KE possessed KL114 capsule type, which is a rare capsule type in *K. pneumoniae* (61) that has been reported recently by Long et al. in drug-resistant human pathogenic *K. quasipneumoniae* strains (40). Isolates 5KE, 6KE, and 14KE harboring the KL66 K-loci have been identified in *K. oxytoca*, which is closely associated with *K. quasipneumoniae* (62). The sharing of these genes among *Klebsiella* species suggests the horizontal gene transfer among these species (63). Moreover, the KL81 and KL146 capsule types were found in 8KE and 10KE respectively. Regarding the prediction of LPS O-antigen gene, three isolates (3KE, 4KE and 14KE) belonged to O3/O3a serotype. Although O3/O3a serotypes pathogenic strains of *K. quasipneumoniae* have been reported, and with the strong adjuvant effect of O3/O3a serotypes, the clinical impact of this serotype is still unknown (59). Isolates 5KE, 6KE and 10KE belonged O12 serotype. In earlier studies, analyses of 573 pathogenic strains of *K. pneumoniae* revealed that 9.2% belonged to O12 serotype (63).

Recently discovered T6SS in *K. pneumoniae* strains plays a role in bacterial warfare and long-term gastrointestinal colonization (64). Although, the presence of T6SS in *K. quasipneumoniae* is low, in this study isolate 8KE was characterized by the presence of 16 types of secretion systems (T6SS-I) and 15 different clustered T6SS-II genes, while 4KE was characterized by the presence of 14 types of T6SS-III, suggesting their virulence and pathogenic activity.

## Conclusion

In summary, we characterized genomes of novel strains of *K. quasipneumoniae* subsp. *similipneumoniae* (*n* = 6) and *K. quasipneumoniae* subsp. *quasipneumoniae* (*n* = 1), harboring different ARGs circulating in drinking water, table surfaces and vegetables in Khartoum markets. The aminoglycoside-modifying enzyme gene [*aph(3”)-Ia*] was harbored in isolate 6KE and was bracketed by two transposable insertion elements IS*5075* and IS*91* transposase. The isolates were identified with key virulence factors occurring in pathogenic *Klebsiella* spp. (CPS and LPS), and possessed a group of other virulence genes such as type 3 fimbriae (*mrkA, mrkB, mrkC, mrkD, mrkF, mrkH*) associated with adherence and biofilm formation. Additionally, the iron uptake aerobactin (*iutA*), salmochelins (*iroE, iroN*), and enterobactin siderophores (*ent* A-F, *feb*-G), *entS, and fes* were identified in all of the isolates except the *iutA* gene which was absent in one isolate (8KE). Such isolates represent a potential risk of being transmitted to humans and can cause hospital or community-acquired infections. We also demonstrated that a large percentage (23%) of *K. pneumoniae* isolates were misidentified; this implies that routine biochemical tests are not enough for species identification and more robust molecular detection methods need to be used in order to improve our understanding which will have a direct impact on improving public health.

## Data availability statement

The datasets presented in this study can be found in online repositories. The names of the repository/repositories and accession number(s) can be found in the article/Supplementary material.

## Ethics statement

This study was approved by the Ethics Committee of the Khartoum State Ministry of Health (Ref: 2/2021).

## Author contributions

Conceptualization: HA and HE. Methodology: HA, EM, and HE. Validation: BL and EM. Formal analysis: BL, IK, and HA. Writing—original draft preparation: IK, OB, SH, IA, HE, and BL. Writing—review and editing: BL and HA. Supervision: SH, HA, and IA. Analysis of fastANI and genome comparison: EM. All authors have read and agreed to the published version of the manuscript.
