# Supplementary material for: Molecular insights into novel environmental strains of Klebsiella quasipneumoniae harboring different antimicrobial-resistance genes
Source: Front Public Health. 2023 Jan 12;10:1068888. doi: 10.3389/fpubh.2022.1068888 (PMC9878601; doi:10.3389/fpubh.2022.1068888)
Supplement: Supplementary file 1 [file Data_Sheet_1.zip › Supplementary File 1_BSL.docx]

Table S1. Comparison of *K. quasipneumoniae* genomes typing and presence of RG

| Strain | ST | cgMLST classification and profile comparison | | |  |  | K locus | O locus | RG | Plasmids |
| --- | --- | --- | --- | --- | --- | --- | --- | --- | --- | --- |
|  |  | Sublineage | Clonal group | cgST | Closest defined cgST(s) | Identity |  |  |  |  |
| 3KE | 5926 | 10719 | 11344 | 11741 |  |  | KL114 | O3/O3a | *fosA, bla_OKP-B-10_, OqxB, OqxA, fosA7, ompK36, ompK37* | IncFIBK |
| 4KE | 4768 | New | New | *128a | 11388 | 63.86% (387/606) | KL114 | O3/O3a | *bla_OKP-A-_3, OqxB, OqxA, fosA6, fosA, ompK36, ompK37* | ColpHAD28, ColpHAD28, ColpVC, IncFIBK, IncFIIpKPX1, IncN, IncR |
| 5KE | 2019 | 10760 | New | *c942 | 11894 | 79.57% (483/607) | KL66 | O12 | *fosA, OqxB, OqxA, bla_OKP-B-2_* | IncFIB_K_ |
| 6KE | 5944 | 10760 | New | *5e89 | 11894 | 78.67% (476/605) | KL66 | O12 | *fosA, OqxB, OqxA, bla_OKP-B-2_, ompK36, ompK37, bla_SHV-1_*, *bla_OXA-1_, aph(3'')-Ib, AAC(6')-Ib-cr, CatB* | IncFIB_K_ |
| 8KE | 5922 | 3599 | New | *708a | 5380 | 85.738% (517/603) | KL81 | K81 | *fosA, bla_OKP-B-10_, OqxB, OqxA* | IncFIB_K_ |
| 10KE | 5945 | 10720 | 11346 | 11743 |  |  | KL146 | O12 | *fosA, bla_OKP-B-14_, OqxB, OqxA, ompK36, ompK37* | NIL |
| 14KE | 5927 | 367 | 11345 | 11742 |  |  | KL166 | O3/O3a | *fosA, bla_OKP-B-14_, OqxB, OqxA, ompK36, ompK37* | Col440I, IncFIB_K_ |
| MGH96 | 3562 | 3562 | 3562 | 5010 |  |  | KL127 | OL103 | OKP-A-3, OqxB, fosA, OqxA | no |
| HKUOPA4 | 3870 | 3870 | 3870 | 4990 |  |  | KL8 | O12 | OKP-A-3, OqxB, fosA, OqxA | no |
| HKUOPL4 | 3870 | 3870 | 3870 | 4990 |  |  | KL8 | O12 | OKP-A-3, OqxB, fosA, OqxA | no |
| ATCC700603 | 489 | 489 | 489 | 4948 |  |  | KL53 | O3/O3a | OKP-A-3, OqxB, fosA, OqxA | no |
| 18A069 | 1118 | 1118 | 1118 | *6b67 | 15 | 99.6683% (601/603) | KL10 | O3/O3a | OKP-A-3, OqxB, fosA, OqxA | no |

Table S2. Alleles differences are described using distance matrices among subspecies of K. quasipneumoniae.

| Name | 10KE | 14KE | 18A069 | 3KE | 4KE | 5KE | 6KE | 8KE | ATCC700603 | HKUOPA4 | HKUOPL4 | MGH96 |
| --- | --- | --- | --- | --- | --- | --- | --- | --- | --- | --- | --- | --- |
| 10KE | 0 | 12932 | 48430 | 12218 | 47430 | 12066 | 12113 | 12645 | 12799 | 12608 | 12608 | 48009 |
| 14KE | 12932 | 0 | 48110 | 10325 | 47133 | 10272 | 10284 | 10214 | 10425 | 10275 | 10275 | 47669 |
| 18A069 | 48430 | 48110 | 0 | 47622 | 11284 | 47514 | 47491 | 48045 | 47900 | 47991 | 47991 | 10238 |
| 3KE | 12218 | 10325 | 47622 | 0 | 46653 | 10085 | 10097 | 10076 | 9204 | 10269 | 10269 | 47206 |
| 4KE | 47430 | 47133 | 11284 | 46653 | 0 | 46484 | 46501 | 47127 | 46859 | 47052 | 47052 | 10368 |
| 5KE | 12066 | 10272 | 47514 | 10085 | 46484 | 0 | 71 | 9323 | 10031 | 9476 | 9476 | 47107 |
| 6KE | 12113 | 10284 | 47491 | 10097 | 46501 | 71 | 0 | 9322 | 10056 | 9512 | 9512 | 47087 |
| 8KE | 12645 | 10214 | 48045 | 10076 | 47127 | 9323 | 9322 | 0 | 10140 | 9504 | 9504 | 47676 |
| ATCC700603 | 12799 | 10425 | 47900 | 9204 | 46859 | 10031 | 10056 | 10140 | 0 | 10282 | 10282 | 47502 |
| HKUOPA4 | 12608 | 10275 | 47991 | 10269 | 47052 | 9476 | 9512 | 9504 | 10282 | 0 | 0 | 47577 |
| HKUOPL4 | 12608 | 10275 | 47991 | 10269 | 47052 | 9476 | 9512 | 9504 | 10282 | 0 | 0 | 47577 |
| MGH96 | 48009 | 47669 | 10238 | 47206 | 10368 | 47107 | 47087 | 47676 | 47502 | 47577 | 47577 | 0 |

Table S3. Efflux pump genes and point mutation in the isolates

| **ID** | **efflux** | **Point mutations** |
| --- | --- | --- |
| 3KE | OKP-B-6, oqxA, Klebsiella pneumoniae KpnG, LptD, CRP, eptB, ArnT, Klebsiella pneumoniae KpnF | acrR: Y114F, V165I |
| 4KE | ArnT, OmpA, CRP, eptB, Klebsiella pneumoniae KpnF, Klebsiella pneumoniae KpnE, oqxA, LptD, Klebsiella pneumoniae KpnG | EF-Tu: R234F |
| 5KE | OKP-B-2, oqxA, Klebsiella pneumoniae KpnG, FosA6, eptB, ArnT, Klebsiella pneumoniae KpnF, CRP, LptD | EF-Tu: R234F, acrR: Y114F, V165I |
| 6KE | OKP-B-2, OmpA, CRP, LptD, eptB, ArnT, oqxA, Klebsiella pneumoniae KpnG | EF-Tu: R234F  acrR: Y114F, V165I |
| 8KE | OKP-B-2, OmpA, CRP, LptD, eptB, ArnT, oqxA, | Nil |
| 10KE | Klebsiella pneumoniae KpnG, oqxA, eptB, LptD, OKP-B-41, CRP, ArnT, OmpA, Klebsiella pneumoniae KpnF | acrR: Y114F, V165I |
| 14KE | ArnT, Klebsiella pneumoniae KpnG, Klebsiella pneumoniae KpnF, LptD, OKP-B-6, oqxA, CRP, eptB | acrR: Y114F, V165I |

Table S4. Predicted virulence factors among the isolates and reference strains (ATCC35657, and MGH96)

| **Adherence** | **Gene** | **3KE** | **4KE** | **5KE** | **6KE** | **8KE** | **10KE** | **14KE** | **ATCC35657** | **MGH96** | **18A069** | **HKUOPLA4** | **HKUOPA4** |
| --- | --- | --- | --- | --- | --- | --- | --- | --- | --- | --- | --- | --- | --- |
| **Adherence** | | | | | | | | | | | | | |
| [Type 3 fimbriae](javascript:void(0)) | [*mrkA*](javascript:void(0)) | [+](javascript:void(0)) | [+](javascript:void(0)) | [+](javascript:void(0)) | [+](javascript:void(0)) | [+](javascript:void(0)) | [+](javascript:void(0)) | [+](javascript:void(0)) | [+](javascript:void(0)) | [+](javascript:void(0)) | [+](javascript:void(0)) | [+](javascript:void(0)) | [+](javascript:void(0)) |
| [Type 3 fimbriae](javascript:void(0)) | [*mrkB*](javascript:void(0)) | [+](javascript:void(0)) | [+](javascript:void(0)) | [+](javascript:void(0)) | [+](javascript:void(0)) | [+](javascript:void(0)) | [+](javascript:void(0)) | [+](javascript:void(0)) | [+](javascript:void(0)) | [+](javascript:void(0)) | [+](javascript:void(0)) | [+](javascript:void(0)) | [+](javascript:void(0)) |
| [Type 3 fimbriae](javascript:void(0)) | [*mrkC*](javascript:void(0)) | [+](javascript:void(0)) | [+](javascript:void(0)) | [+](javascript:void(0)) | [+](javascript:void(0)) | [+](javascript:void(0)) | [+](javascript:void(0)) | [+](javascript:void(0)) | [+](javascript:void(0)) | [+](javascript:void(0)) | [+](javascript:void(0)) | [+](javascript:void(0)) | [+](javascript:void(0)) |
| [Type 3 fimbriae](javascript:void(0)) | [*mrkD*](javascript:void(0)) | [+](javascript:void(0)) | [+](javascript:void(0)) | [+](javascript:void(0)) | [+](javascript:void(0)) | [+](javascript:void(0)) | [+](javascript:void(0)) | [+](javascript:void(0)) | [+](javascript:void(0)) | [+](javascript:void(0)) | [+](javascript:void(0)) | [+](javascript:void(0)) | [+](javascript:void(0)) |
| [Type 3 fimbriae](javascript:void(0)) | [*mrkF*](javascript:void(0)) | [+](javascript:void(0)) | [+](javascript:void(0)) | [+](javascript:void(0)) | [+](javascript:void(0)) | [+](javascript:void(0)) | [+](javascript:void(0)) | [+](javascript:void(0)) | [+](javascript:void(0)) | [+](javascript:void(0)) | [+](javascript:void(0)) | [+](javascript:void(0)) | [+](javascript:void(0)) |
| [Type 3 fimbriae](javascript:void(0)) | [*mrkH*](javascript:void(0)) | [+](javascript:void(0)) | [+](javascript:void(0)) | [+](javascript:void(0)) | [+](javascript:void(0)) | [+](javascript:void(0)) | [+](javascript:void(0)) | [+](javascript:void(0)) | [+](javascript:void(0)) | [+](javascript:void(0)) | [+](javascript:void(0)) | [+](javascript:void(0)) | [+](javascript:void(0)) |
| [Type 3 fimbriae](javascript:void(0)) | [*mrkI*](javascript:void(0)) | [+](javascript:void(0)) | [+](javascript:void(0)) | [+](javascript:void(0)) | [+](javascript:void(0)) | [+](javascript:void(0)) | - | [+](javascript:void(0)) | [+](javascript:void(0)) | [+](javascript:void(0)) | [+](javascript:void(0)) | [+](javascript:void(0)) | [+](javascript:void(0)) |
| [Type 3 fimbriae](javascript:void(0)) | [*mrkJ*](javascript:void(0)) | [+](javascript:void(0)) | [+](javascript:void(0)) | [+](javascript:void(0)) | - | [+](javascript:void(0)) | [+](javascript:void(0)) | [+](javascript:void(0)) | [+](javascript:void(0)) | [+](javascript:void(0)) | [+](javascript:void(0)) | [+](javascript:void(0)) | [+](javascript:void(0)) |
| [Type I fimbriae](javascript:void(0)) | [*fimA*](javascript:void(0)) | [+](javascript:void(0)) | - | [+](javascript:void(0)) | [+](javascript:void(0)) | - | [+](javascript:void(0)) | [+](javascript:void(0)) | [+](javascript:void(0)) | [+](javascript:void(0)) | [+](javascript:void(0)) | [+](javascript:void(0)) | [+](javascript:void(0)) |
| [Type I fimbriae](javascript:void(0)) | [*fimB*](javascript:void(0)) | [+](javascript:void(0)) | [+](javascript:void(0)) | - | [+](javascript:void(0)) | - | [+](javascript:void(0)) | [+](javascript:void(0)) | [+](javascript:void(0)) | [+](javascript:void(0)) | [+](javascript:void(0)) | [+](javascript:void(0)) | [+](javascript:void(0)) |
| [Type I fimbriae](javascript:void(0)) | [*fimC*](javascript:void(0)) | [+](javascript:void(0)) | [+](javascript:void(0)) | [+](javascript:void(0)) | [+](javascript:void(0)) | - | [+](javascript:void(0)) | [+](javascript:void(0)) | [+](javascript:void(0)) | [+](javascript:void(0)) | [+](javascript:void(0)) | [+](javascript:void(0)) | [+](javascript:void(0)) |
| [Type I fimbriae](javascript:void(0)) | [*fimD*](javascript:void(0)) | [+](javascript:void(0)) | [+](javascript:void(0)) | [+](javascript:void(0)) | [+](javascript:void(0)) | [+](javascript:void(0)) | [+](javascript:void(0)) | [+](javascript:void(0)) | [+](javascript:void(0)) | [+](javascript:void(0)) | [+](javascript:void(0)) | [+](javascript:void(0)) | [+](javascript:void(0)) |
| [Type I fimbriae](javascript:void(0)) | [*fimE*](javascript:void(0)) | [+](javascript:void(0)) | [+](javascript:void(0)) | [+](javascript:void(0)) | [+](javascript:void(0)) | - | [+](javascript:void(0)) | [+](javascript:void(0)) | [+](javascript:void(0)) | [+](javascript:void(0)) | [+](javascript:void(0)) | [+](javascript:void(0)) | [+](javascript:void(0)) |
| [Type I fimbriae](javascript:void(0)) | [*fimF*](javascript:void(0)) | [+](javascript:void(0)) | - | [+](javascript:void(0)) | [+](javascript:void(0)) | - | [+](javascript:void(0)) | [+](javascript:void(0)) | [+](javascript:void(0)) | [+](javascript:void(0)) | [+](javascript:void(0)) | [+](javascript:void(0)) | [+](javascript:void(0)) |
| [Type I fimbriae](javascript:void(0)) | [*fimG*](javascript:void(0)) | [+](javascript:void(0)) | [+](javascript:void(0)) | [+](javascript:void(0)) | [+](javascript:void(0)) | - | [+](javascript:void(0)) | [+](javascript:void(0)) | [+](javascript:void(0)) | [+](javascript:void(0)) | [+](javascript:void(0)) | [+](javascript:void(0)) | [+](javascript:void(0)) |
| [Type I fimbriae](javascript:void(0)) | [*fimH*](javascript:void(0)) | [+](javascript:void(0)) | [+](javascript:void(0)) | [+](javascript:void(0)) | [+](javascript:void(0)) | - | [+](javascript:void(0)) | [+](javascript:void(0)) | [+](javascript:void(0)) | [+](javascript:void(0)) | [+](javascript:void(0)) | [+](javascript:void(0)) | [+](javascript:void(0)) |
| [Type I fimbriae](javascript:void(0)) | [*fimI*](javascript:void(0)) | [+](javascript:void(0)) | [+](javascript:void(0)) | [+](javascript:void(0)) | [+](javascript:void(0)) | - | [+](javascript:void(0)) | [+](javascript:void(0)) | [+](javascript:void(0)) | [+](javascript:void(0)) | [+](javascript:void(0)) | [+](javascript:void(0)) | [+](javascript:void(0)) |
| [Type I fimbriae](javascript:void(0)) | [*fimK*](javascript:void(0)) | [+](javascript:void(0)) | [+](javascript:void(0)) | [+](javascript:void(0)) | [+](javascript:void(0)) | - | [+](javascript:void(0)) | [+](javascript:void(0)) | [+](javascript:void(0)) | [+](javascript:void(0)) | - | [+](javascript:void(0)) | [+](javascript:void(0)) |
| **Antiphagocytosis (1 Item)** | | | | | | | | | | | | | |
|  | Capsule | [+](javascript:void(0)) | [+](javascript:void(0)) | [+](javascript:void(0)) | [+](javascript:void(0)) | [+](javascript:void(0)) | [+](javascript:void(0)) | [+](javascript:void(0)) | [+](javascript:void(0)) | [+](javascript:void(0)) | [+](javascript:void(0)) | [+](javascript:void(0)) | [+](javascript:void(0)) |
|  |  | [+](javascript:void(0)) |  | [+](javascript:void(0)) | [+](javascript:void(0)) | [+](javascript:void(0)) | [+](javascript:void(0)) | [+](javascript:void(0)) | [+](javascript:void(0)) | [+](javascript:void(0)) | [+](javascript:void(0)) | [+](javascript:void(0)) | [+](javascript:void(0)) |
|  |  | [+](javascript:void(0)) | [+](javascript:void(0)) | [+](javascript:void(0)) | [+](javascript:void(0)) | [+](javascript:void(0)) | [+](javascript:void(0)) | [+](javascript:void(0)) | [+](javascript:void(0)) | [+](javascript:void(0)) | [+](javascript:void(0)) | [+](javascript:void(0)) | [+](javascript:void(0)) |
|  |  | [+](javascript:void(0)) |  | [+](javascript:void(0)) | [+](javascript:void(0)) | [+](javascript:void(0)) | [+](javascript:void(0)) | [+](javascript:void(0)) | [+](javascript:void(0)) | [+](javascript:void(0)) | [+](javascript:void(0)) | [+](javascript:void(0)) | [+](javascript:void(0)) |
|  |  | [+](javascript:void(0)) |  | [+](javascript:void(0)) | [+](javascript:void(0)) | [+](javascript:void(0)) | [+](javascript:void(0)) | [+](javascript:void(0)) | [+](javascript:void(0)) | [+](javascript:void(0)) | [+](javascript:void(0)) | [+](javascript:void(0)) | [+](javascript:void(0)) |
|  |  | [+](javascript:void(0)) | [+](javascript:void(0)) | [+](javascript:void(0)) | [+](javascript:void(0)) | [+](javascript:void(0)) | [+](javascript:void(0)) | [+](javascript:void(0)) | [+](javascript:void(0)) | [+](javascript:void(0)) | [+](javascript:void(0)) | [+](javascript:void(0)) | [+](javascript:void(0)) |
|  |  | [+](javascript:void(0)) | [+](javascript:void(0)) | [+](javascript:void(0)) | [+](javascript:void(0)) | [+](javascript:void(0)) | [+](javascript:void(0)) | [+](javascript:void(0)) | [+](javascript:void(0)) | [+](javascript:void(0)) | [+](javascript:void(0)) | [+](javascript:void(0)) | [+](javascript:void(0)) |
|  |  | [+](javascript:void(0)) | [+](javascript:void(0)) | [+](javascript:void(0)) | [+](javascript:void(0)) | [+](javascript:void(0)) | [+](javascript:void(0)) | [+](javascript:void(0)) | [+](javascript:void(0)) | [+](javascript:void(0)) | [+](javascript:void(0)) | [+](javascript:void(0)) | [+](javascript:void(0)) |
|  |  | [+](javascript:void(0)) | [+](javascript:void(0)) | [+](javascript:void(0)) | [+](javascript:void(0)) | [+](javascript:void(0)) | [+](javascript:void(0)) | [+](javascript:void(0)) | [+](javascript:void(0)) | [+](javascript:void(0)) | [+](javascript:void(0)) | [+](javascript:void(0)) | [+](javascript:void(0)) |
|  |  | [+](javascript:void(0)) | [+](javascript:void(0)) | [+](javascript:void(0)) | [+](javascript:void(0)) | [+](javascript:void(0)) | [+](javascript:void(0)) | [+](javascript:void(0)) | [+](javascript:void(0)) | [+](javascript:void(0)) | [+](javascript:void(0)) | [+](javascript:void(0)) | [+](javascript:void(0)) |
|  |  | - | [+](javascript:void(0)) | [+](javascript:void(0)) | [+](javascript:void(0)) | - | [+](javascript:void(0)) | [+](javascript:void(0)) | [+](javascript:void(0)) | [+](javascript:void(0)) | [+](javascript:void(0)) | [+](javascript:void(0)) | [+](javascript:void(0)) |
|  |  | - | [+](javascript:void(0)) | [+](javascript:void(0)) | [+](javascript:void(0)) | - | [+](javascript:void(0)) | [+](javascript:void(0)) | [+](javascript:void(0)) | [+](javascript:void(0)) | [+](javascript:void(0)) | [+](javascript:void(0)) | [+](javascript:void(0)) |
|  |  | - | [+](javascript:void(0)) | [+](javascript:void(0)) | [+](javascript:void(0)) | - | [+](javascript:void(0)) | [+](javascript:void(0)) | [+](javascript:void(0)) | [+](javascript:void(0)) | [+](javascript:void(0)) | [+](javascript:void(0)) | [+](javascript:void(0)) |
|  |  | - | [+](javascript:void(0)) | [+](javascript:void(0)) | [+](javascript:void(0)) | - | [+](javascript:void(0)) | [+](javascript:void(0)) | [+](javascript:void(0)) | [+](javascript:void(0)) | [+](javascript:void(0)) | - | - |
|  |  | - | [+](javascript:void(0)) | [+](javascript:void(0)) | [+](javascript:void(0)) | - | [+](javascript:void(0)) | [+](javascript:void(0)) | [+](javascript:void(0)) | - | - | - | - |
|  |  | - | [+](javascript:void(0)) | [+](javascript:void(0)) | [+](javascript:void(0)) | - | [+](javascript:void(0)) | [+](javascript:void(0)) | [+](javascript:void(0)) | - | - | - | - |
|  |  | - | - | - | [+](javascript:void(0)) |  | [+](javascript:void(0)) | [+](javascript:void(0)) | - | - | - | - | - |
|  |  | - | - | - |  |  |  | [+](javascript:void(0)) | - | - | - | - | - |
| **Efflux pump (2 Items)** | | | | | | | | | | | | | |
| [AcrAB](javascript:void(0)) | *acrA* | [+](javascript:void(0)) | [+](javascript:void(0)) | [+](javascript:void(0)) | [+](javascript:void(0)) | [+](javascript:void(0)) | [+](javascript:void(0)) | [+](javascript:void(0)) | [+](javascript:void(0)) | [+](javascript:void(0)) | [+](javascript:void(0)) | [+](javascript:void(0)) | [+](javascript:void(0)) |
| AcrAB | *acrB* | [+](javascript:void(0)) | [+](javascript:void(0)) | [+](javascript:void(0)) | [+](javascript:void(0)) | [+](javascript:void(0)) | [+](javascript:void(0)) | [+](javascript:void(0)) | [+](javascript:void(0)) | [+](javascript:void(0)) | [+](javascript:void(0)) | [+](javascript:void(0)) | [+](javascript:void(0)) |
|  |  | [+](javascript:void(0)) | - | [+](javascript:void(0)) | [+](javascript:void(0)) | [+](javascript:void(0)) | [+](javascript:void(0)) | [+](javascript:void(0)) | [+](javascript:void(0)) | [+](javascript:void(0)) |  |  |  |
|  |  | [+](javascript:void(0)) | - | - | - | - | - | - | - | - |  |  |  |
| **Iron uptake (34 Items)** | | | | | | | | | | | | | |
| Aerobactin | *iucA* | - | - | - | - | - | - | - | - | - | - | - | - |
| Aerobactin | *iucB* | - | - | - | - | - | - | - | - | - | - | - | - |
| [Aerobactin](javascript:void(0)) | *iucC* | - | - | - | - | - | - | - | - | - | - | - | - |
| [Aerobactin](javascript:void(0)) | *iucD* | - | - | - | - | - | - | - | - | - | - | - | - |
| [Aerobactin](javascript:void(0)) | *iutA* | [+](javascript:void(0)) | [+](javascript:void(0)) | [+](javascript:void(0)) | [+](javascript:void(0)) | [+](javascript:void(0)) | - | [+](javascript:void(0)) | [+](javascript:void(0)) | [+](javascript:void(0)) | [+](javascript:void(0)) | [+](javascript:void(0)) | [+](javascript:void(0)) |
| [Ent siderophore](javascript:void(0)) | *entA* | [+](javascript:void(0)) | [+](javascript:void(0)) | [+](javascript:void(0)) | [+](javascript:void(0)) | [+](javascript:void(0)) | [+](javascript:void(0)) | [+](javascript:void(0)) | [+](javascript:void(0)) | [+](javascript:void(0)) | [+](javascript:void(0)) | [+](javascript:void(0)) | [+](javascript:void(0)) |
| [Ent siderophore](javascript:void(0)) | *entB* | [+](javascript:void(0)) | [+](javascript:void(0)) | [+](javascript:void(0)) | [+](javascript:void(0)) | [+](javascript:void(0)) | [+](javascript:void(0)) | [+](javascript:void(0)) | [+](javascript:void(0)) | [+](javascript:void(0)) | [+](javascript:void(0)) | [+](javascript:void(0)) | [+](javascript:void(0)) |
| Ent siderophore | *entC* | [+](javascript:void(0)) | [+](javascript:void(0)) | [+](javascript:void(0)) | [+](javascript:void(0)) | [+](javascript:void(0)) | [+](javascript:void(0)) | [+](javascript:void(0)) | [+](javascript:void(0)) | [+](javascript:void(0)) | [+](javascript:void(0)) | [+](javascript:void(0)) | [+](javascript:void(0)) |
| Ent siderophore | *entD* | [+](javascript:void(0)) | [+](javascript:void(0)) | [+](javascript:void(0)) | - | [+](javascript:void(0)) | [+](javascript:void(0)) | [+](javascript:void(0)) | [+](javascript:void(0)) | [+](javascript:void(0)) | [+](javascript:void(0)) | [+](javascript:void(0)) | [+](javascript:void(0)) |
| Ent siderophore | *entE* | [+](javascript:void(0)) | [+](javascript:void(0)) | [+](javascript:void(0)) | [+](javascript:void(0)) | [+](javascript:void(0)) | [+](javascript:void(0)) | [+](javascript:void(0)) | [+](javascript:void(0)) | [+](javascript:void(0)) | [+](javascript:void(0)) | [+](javascript:void(0)) | [+](javascript:void(0)) |
| Ent siderophore | *entF* | [+](javascript:void(0)) | [+](javascript:void(0)) | [+](javascript:void(0)) | [+](javascript:void(0)) | [+](javascript:void(0)) | [+](javascript:void(0)) | [+](javascript:void(0)) | [+](javascript:void(0)) | [+](javascript:void(0)) | [+](javascript:void(0)) | [+](javascript:void(0)) | [+](javascript:void(0)) |
| Ent siderophore | *entS* | [+](javascript:void(0)) | [+](javascript:void(0)) | [+](javascript:void(0)) | [+](javascript:void(0)) | [+](javascript:void(0)) | [+](javascript:void(0)) | [+](javascript:void(0)) | [+](javascript:void(0)) | [+](javascript:void(0)) | [+](javascript:void(0)) | [+](javascript:void(0)) | [+](javascript:void(0)) |
| Ent siderophore | *fepA* | [+](javascript:void(0)) | [+](javascript:void(0)) | [+](javascript:void(0)) | [+](javascript:void(0)) | [+](javascript:void(0)) | [+](javascript:void(0)) | [+](javascript:void(0)) | [+](javascript:void(0)) | [+](javascript:void(0)) | [+](javascript:void(0)) | [+](javascript:void(0)) | [+](javascript:void(0)) |
|  |  | [+](javascript:void(0)) | [+](javascript:void(0)) | [+](javascript:void(0)) | [+](javascript:void(0)) | [+](javascript:void(0)) | [+](javascript:void(0)) | [+](javascript:void(0)) | [+](javascript:void(0)) | [+](javascript:void(0)) | [+](javascript:void(0)) | [+](javascript:void(0)) | [+](javascript:void(0)) |
| Ent siderophore | *fepB* | [+](javascript:void(0)) | [+](javascript:void(0)) | [+](javascript:void(0)) | [+](javascript:void(0)) | [+](javascript:void(0)) | [+](javascript:void(0)) | [+](javascript:void(0)) | [+](javascript:void(0)) | [+](javascript:void(0)) | [+](javascript:void(0)) | [+](javascript:void(0)) | [+](javascript:void(0)) |
| Ent siderophore | *fepC* | [+](javascript:void(0)) | [+](javascript:void(0)) | [+](javascript:void(0)) | [+](javascript:void(0)) | [+](javascript:void(0)) | [+](javascript:void(0)) | [+](javascript:void(0)) | [+](javascript:void(0)) | [+](javascript:void(0)) | [+](javascript:void(0)) | [+](javascript:void(0)) | [+](javascript:void(0)) |
| Ent siderophore | *fepD* | [+](javascript:void(0)) | [+](javascript:void(0)) | [+](javascript:void(0)) | [+](javascript:void(0)) | [+](javascript:void(0)) | [+](javascript:void(0)) | [+](javascript:void(0)) | [+](javascript:void(0)) | [+](javascript:void(0)) | [+](javascript:void(0)) | [+](javascript:void(0)) | [+](javascript:void(0)) |
| Ent siderophore | *fepG* | [+](javascript:void(0)) | [+](javascript:void(0)) | [+](javascript:void(0)) | [+](javascript:void(0)) | [+](javascript:void(0)) | [+](javascript:void(0)) | [+](javascript:void(0)) | [+](javascript:void(0)) | [+](javascript:void(0)) | [+](javascript:void(0)) | [+](javascript:void(0)) | [+](javascript:void(0)) |
| Ent siderophore | *fes* | [+](javascript:void(0)) |  | [+](javascript:void(0)) | [+](javascript:void(0)) | [+](javascript:void(0)) | [+](javascript:void(0)) | [+](javascript:void(0)) | [+](javascript:void(0)) | [+](javascript:void(0)) | [+](javascript:void(0)) | [+](javascript:void(0)) | [+](javascript:void(0)) |
| Salmochelin | *IroB* | - | - | - | - | - | - | - | - | - | - | - | - |
| Salmochelin | *iroC* | - | - | - | - | - | - | - | - | - | - | - | - |
| Salmochelin | *iroD* | - | - | - | - | - | - | - | - | - | - | - | - |
| [Salmochelin](javascript:void(0)) | *iroE* | [+](javascript:void(0)) | [+](javascript:void(0)) | [+](javascript:void(0)) | [+](javascript:void(0)) | [+](javascript:void(0)) | [+](javascript:void(0)) | [+](javascript:void(0)) | [+](javascript:void(0)) | [+](javascript:void(0)) | [+](javascript:void(0)) | [+](javascript:void(0)) | [+](javascript:void(0)) |
| [Salmochelin](javascript:void(0)) | *iroN* | [+](javascript:void(0)) | [+](javascript:void(0)) | [+](javascript:void(0)) | [+](javascript:void(0)) | [+](javascript:void(0)) | [+](javascript:void(0)) | [+](javascript:void(0)) | [+](javascript:void(0)) | - | - | [+](javascript:void(0)) | [+](javascript:void(0)) |
| [Yersiniabactin](javascript:void(0)) | *fyuA* | - |  | - | - | - | - | - | - | - | - | - | - |
| Yersiniabactin | *irp1* | - |  | - | - | - | - | - | - | - | - | - | - |
| Yersiniabactin | *irp2* | - |  | - | - | - | - | - | - | - | - | - | - |
| [Yersiniabactin](javascript:void(0)) | *ybtA* | - |  | - | - | - | - | - | - | - | - | - | - |
| [Yersiniabactin](javascript:void(0)) | *ybtE* | - |  | - | - | - | - | - | - | - | - | - | - |
| [Yersiniabactin](javascript:void(0)) | *ybtP* | - |  | - | - | - | - | - | - | - | - | - | - |
| [Yersiniabactin](javascript:void(0)) | *ybtQ* | - |  | - | - | - | - | - | - | - | - | - | - |
| [Yersiniabactin](javascript:void(0)) | *ybtS* | - |  | - | - | - | - | - | - | - | - | - | - |
| [Yersiniabactin](javascript:void(0)) | *ybtT* | - |  | - | - | - | - | - | - | - | - | - | - |
| [Yersiniabactin](javascript:void(0)) | *ybtU* | - |  | - | - | - | - | - | - | - | - | - | - |
| [Yersiniabactin](javascript:void(0)) | *ybtX* | - |  | - | - | - | - | - | - | - | - | - | - |
| **Nutritional factor (6 Items)** | | | | | | | | | | | | | |
| [Allantoin utilization](javascript:void(0)) | *allA* | - | - | - | - | - | - | - | - | - | - | - | - |
| [Allantoin utilization](javascript:void(0)) | *allB* | - | - | - | - | - | - | - | - | - | - | - | - |
| [Allantoin utilization](javascript:void(0)) | *allC* | - | - | - | - | - | - | - | - | - | - | - | - |
| [Allantoin utilization](javascript:void(0)) | *allD* | - | - | - | - | - | - | - | - | - | - | - | - |
| [Allantoin utilization](javascript:void(0)) | *allR* | - | - | - | - | - | - | - | - | - | - | - | - |
| [Allantoin utilization](javascript:void(0)) | *allS* | - | - | - | - | - | - | - | - | - | - | - | - |
| **Regulation (3 Items)** | | | | | | | | | | | | | |
| [RcsAB](javascript:void(0)) | *rcsA* | [+](javascript:void(0)) | [+](javascript:void(0)) | [+](javascript:void(0)) | [+](javascript:void(0)) | [+](javascript:void(0)) | [+](javascript:void(0)) | [+](javascript:void(0)) | [+](javascript:void(0)) | [+](javascript:void(0)) | [+](javascript:void(0)) | [+](javascript:void(0)) | [+](javascript:void(0)) |
| [RcsAB](javascript:void(0)) | *rcsB* | [+](javascript:void(0)) | [+](javascript:void(0)) | [+](javascript:void(0)) | [+](javascript:void(0)) | [+](javascript:void(0)) | [+](javascript:void(0)) | [+](javascript:void(0)) | [+](javascript:void(0)) | [+](javascript:void(0)) | [+](javascript:void(0)) | [+](javascript:void(0)) | [+](javascript:void(0)) |
| [RmpA](javascript:void(0)) | *rmpA* | - | - | - | - | - | - | - | - | - | - | - | - |
| **Secretion system (46 Items)** | | | | | | | | | | | | | |
| T6SS-I | *-* | - | - | - | - | [+](javascript:void(0)) | - | - |  | [+](javascript:void(0)) |  | - | - |
| [T6SS-I](javascript:void(0)) | *-* | - | - | - | - | [+](javascript:void(0)) | - | - |  | [+](javascript:void(0)) |  | - | - |
| [T6SS-I](javascript:void(0)) | *-* | - | - | - | - | [+](javascript:void(0)) | - | - |  | [+](javascript:void(0)) |  | - | - |
| [T6SS-I](javascript:void(0)) | *clpV/tssH* | - | - | - | - | [+](javascript:void(0)) | - | - | [+](javascript:void(0)) | [+](javascript:void(0)) | [+](javascript:void(0)) | - | - |
| [T6SS-I](javascript:void(0)) | *dotU/tssL* | - | - | - | - | [+](javascript:void(0)) | - | - | [+](javascript:void(0)) | [+](javascript:void(0)) | [+](javascript:void(0)) | - | - |
| [T6SS-I](javascript:void(0)) | *hcp/tssD* | - | - | - | - | [+](javascript:void(0)) | - | - | [+](javascript:void(0)) | [+](javascript:void(0)) | [+](javascript:void(0)) | - | - |
| [T6SS-I](javascript:void(0)) | *icmF/tssM* | - | - | - | - | [+](javascript:void(0)) | - | - | [+](javascript:void(0)) | [+](javascript:void(0)) | [+](javascript:void(0)) | - | - |
| [T6SS-I](javascript:void(0)) | *impA/tssA* | - | - | - | - | - | - | - | [+](javascript:void(0)) | - | - | - | - |
| [T6SS-I](javascript:void(0)) | *ompA* | - | - | - | - | [+](javascript:void(0)) | - | - | [+](javascript:void(0)) | [+](javascript:void(0)) | [+](javascript:void(0)) | - | - |
| [T6SS-I](javascript:void(0)) | *sciN/tssJ* | - | - | - | - | [+](javascript:void(0)) | - | - | [+](javascript:void(0)) | [+](javascript:void(0)) | [+](javascript:void(0)) | - | - |
| [T6SS-I](javascript:void(0)) | *tle1* | - | - | - | - | - | - | - | - | - | - | - | - |
| [T6SS-I](javascript:void(0)) | *tli1* | - | - | - | - | - | - | - | - | [+](javascript:void(0)) | - | - | - |
| [T6SS-I](javascript:void(0)) | *tssF* | - | - | - | - | [+](javascript:void(0)) | - | - | [+](javascript:void(0)) | [+](javascript:void(0)) | [+](javascript:void(0)) | - | - |
| [T6SS-I](javascript:void(0)) | *tssG* | - | - | - | - | [+](javascript:void(0)) | - | - | [+](javascript:void(0)) | [+](javascript:void(0)) | [+](javascript:void(0)) | - | - |
| [T6SS-I](javascript:void(0)) | *vasE/tssK* | - | - | - | - | [+](javascript:void(0)) | - | - | [+](javascript:void(0)) | [+](javascript:void(0)) | [+](javascript:void(0)) | - | - |
| [T6SS-I](javascript:void(0)) | *vgrG/tssI* | - | - | - | - | [+](javascript:void(0)) | - | - | [+](javascript:void(0)) | [+](javascript:void(0)) | [+](javascript:void(0)) | - | - |
| [T6SS-I](javascript:void(0)) | *vipA/tssB* | - | - | - | - | [+](javascript:void(0)) | - | - | [+](javascript:void(0)) | [+](javascript:void(0)) | [+](javascript:void(0)) | - | - |
| [T6SS-I](javascript:void(0)) | *vipB/tssC* | - | - | - | - | [+](javascript:void(0)) | - | - | [+](javascript:void(0)) | [+](javascript:void(0)) | [+](javascript:void(0)) | - | - |
| [T6SS-II](https://ncbi.nlm.nih.gov/pubmed/?term=20932940) | *clpV* | [+](javascript:void(0)) | [+](javascript:void(0)) | [+](javascript:void(0)) | [+](javascript:void(0)) | [+](javascript:void(0)) | [+](javascript:void(0)) | [+](javascript:void(0)) | [+](javascript:void(0)) | [+](javascript:void(0)) | [+](javascript:void(0)) | [+](javascript:void(0)) | [+](javascript:void(0)) |
| [T6SS-II](https://ncbi.nlm.nih.gov/pubmed/?term=20932940) | *dotU* | - | - | - | - |  | - | - | - | - | - | - | - |
| [T6SS-II](https://ncbi.nlm.nih.gov/pubmed/?term=20932940) | *icmF* | - | - | - | - | - | - | - | - | - | - | - | - |
| T6SS-II | *impF* | - | - | - | - | - | - | - | - | - | - | - | - |
| [T6SS-II](https://ncbi.nlm.nih.gov/pubmed/?term=20932940) | *impH* | - | - | - | - | - | - | - | - | - | - | - | - |
| [T6SS-II](https://ncbi.nlm.nih.gov/pubmed/?term=20932940) | *impJ* | - | - | - | - | - | - | - | - | - | - | - | - |
| [T6SS-II](https://ncbi.nlm.nih.gov/pubmed/?term=20932940) | *ompA* | - | - | - | - | - | - | - | - | - | - | - | - |
| [T6SS-II](https://ncbi.nlm.nih.gov/pubmed/?term=20932940) | *sciN* | - | - | - | - | - | - | - | - | - | - | - | - |
| [T6SS-II](https://ncbi.nlm.nih.gov/pubmed/?term=20932940) | *vasA/impG* | - | - | - | - | - | - | - | - | - | - | - | - |
| [T6SS-II](https://ncbi.nlm.nih.gov/pubmed/?term=20932940) | *vgrG* | - | - | - | - | - | - | - | - | - | - | - | - |
| [T6SS-III](https://ncbi.nlm.nih.gov/pubmed/?term=20932940) | *-* | - | - | - | - | - | - | - | - | - | - | - | - |
| [T6SS-III](https://ncbi.nlm.nih.gov/pubmed/?term=20932940) | - | - | - | - | - | - | - | - | - | [+](javascript:void(0)) | - | - | - |
| [T6SS-III](https://ncbi.nlm.nih.gov/pubmed/?term=20932940) | - | - | - | - | - | - | - | - | - | - | - | - | - |
| [T6SS-III](https://ncbi.nlm.nih.gov/pubmed/?term=20932940) | - | - | - | - | - | - | - | - | - | - | - | - | - |
| [T6SS-III](https://ncbi.nlm.nih.gov/pubmed/?term=20932940) | - | - | - | - | - | - | - | - | - | - | - | - | - |
| [T6SS-III](https://ncbi.nlm.nih.gov/pubmed/?term=20932940) | - | [+](javascript:void(0)) | [+](javascript:void(0)) | [+](javascript:void(0)) | [+](javascript:void(0)) | [+](javascript:void(0)) | [+](javascript:void(0)) | [+](javascript:void(0)) | [+](javascript:void(0)) | [+](javascript:void(0)) | - | [+](javascript:void(0)) | [+](javascript:void(0)) |
| [T6SS-III](https://ncbi.nlm.nih.gov/pubmed/?term=20932940) | - | [+](javascript:void(0)) | [+](javascript:void(0)) | [+](javascript:void(0)) | [+](javascript:void(0)) | [+](javascript:void(0)) | [+](javascript:void(0)) | [+](javascript:void(0)) | [+](javascript:void(0)) | [+](javascript:void(0)) | - | [+](javascript:void(0)) | [+](javascript:void(0)) |
| [T6SS-III](https://ncbi.nlm.nih.gov/pubmed/?term=20932940) | *dotU* | - | [+](javascript:void(0)) | - | - | - | - | - | [+](javascript:void(0)) | [+](javascript:void(0)) | [-](javascript:void(0)) | [-](javascript:void(0)) | [-](javascript:void(0)) |
| T6SS-III | *icmF* | - | [+](javascript:void(0)) | - | - | - | - | - | [+](javascript:void(0)) | [+](javascript:void(0)) | [+](javascript:void(0)) | [-](javascript:void(0)) | [-](javascript:void(0)) |
| T6SS-III | *impA* | - | [+](javascript:void(0)) | - | - | [+](javascript:void(0)) | - | - | [+](javascript:void(0)) | [+](javascript:void(0)) | [+](javascript:void(0)) | [+](javascript:void(0)) | [+](javascript:void(0)) |
| [T6SS-III](https://ncbi.nlm.nih.gov/pubmed/?term=20932940) | *impF* | - | [+](javascript:void(0)) | - | - | [+](javascript:void(0)) | - | - | [+](javascript:void(0)) | [+](javascript:void(0)) | [+](javascript:void(0)) | [+](javascript:void(0)) | [+](javascript:void(0)) |
| [T6SS-III](https://ncbi.nlm.nih.gov/pubmed/?term=20932940) | *impG* | - | [+](javascript:void(0)) | - | - | - | - | - | [+](javascript:void(0)) | [+](javascript:void(0)) | [+](javascript:void(0)) | [-](javascript:void(0)) | [-](javascript:void(0)) |
| [T6SS-III](https://ncbi.nlm.nih.gov/pubmed/?term=20932940) | *impH* | - | [+](javascript:void(0)) | - | - | - | - | - | [+](javascript:void(0)) | [+](javascript:void(0)) | [+](javascript:void(0)) | [-](javascript:void(0)) | [-](javascript:void(0)) |
| [T6SS-III](https://ncbi.nlm.nih.gov/pubmed/?term=20932940) | *impJ* | - | [+](javascript:void(0)) | - | - | - | - | - | [+](javascript:void(0)) | [+](javascript:void(0)) | [+](javascript:void(0)) | [-](javascript:void(0)) | [-](javascript:void(0)) |
| [T6SS-III](https://ncbi.nlm.nih.gov/pubmed/?term=20932940) | *lysM* | - | - | - | - | - | - | - | - | - | - | [-](javascript:void(0)) | [-](javascript:void(0)) |
| [T6SS-III](https://ncbi.nlm.nih.gov/pubmed/?term=20932940) | *ompA* | - | [+](javascript:void(0)) | - | - | - | - | - | [+](javascript:void(0)) | [+](javascript:void(0)) | [+](javascript:void(0)) | [-](javascript:void(0)) | [-](javascript:void(0)) |
| [T6SS-III](https://ncbi.nlm.nih.gov/pubmed/?term=20932940) | *sciN* | - | [+](javascript:void(0)) | - | - | - | - | - | [+](javascript:void(0)) | [+](javascript:void(0)) | [+](javascript:void(0)) | [-](javascript:void(0)) | [-](javascript:void(0)) |
| [T6SS-III](https://ncbi.nlm.nih.gov/pubmed/?term=20932940) | *vgrG* | - | [+](javascript:void(0)) | - | - | - | - | - | [+](javascript:void(0)) | [+](javascript:void(0)) | [+](javascript:void(0)) | [-](javascript:void(0)) | [-](javascript:void(0)) |
| **Serum resistance** | | | | | | | | | | | | | |
| LPS rfb locus | - | [+](javascript:void(0)) | [+](javascript:void(0)) | - | - | [+](javascript:void(0)) | - | [+](javascript:void(0)) | [+](javascript:void(0)) | - | [+](javascript:void(0)) | [+](javascript:void(0)) |  |
|  |  | [+](javascript:void(0)) | [+](javascript:void(0)) | [+](javascript:void(0)) | [+](javascript:void(0)) | [+](javascript:void(0)) | [+](javascript:void(0)) | [+](javascript:void(0)) | [+](javascript:void(0)) | - | [+](javascript:void(0)) | [+](javascript:void(0)) | [+](javascript:void(0)) |
|  |  | [+](javascript:void(0)) | [+](javascript:void(0)) | - | - | - | - | [+](javascript:void(0)) | [+](javascript:void(0)) | - | [+](javascript:void(0)) | - | [+](javascript:void(0)) |
|  |  | [+](javascript:void(0)) | [+](javascript:void(0)) | - | - | - | - | [+](javascript:void(0)) | [+](javascript:void(0)) | - | [+](javascript:void(0)) | - | - |
|  |  | [+](javascript:void(0)) | [+](javascript:void(0)) | - | - | - | - | [+](javascript:void(0)) | [+](javascript:void(0)) | - | [+](javascript:void(0)) | - | - |
|  |  | [+](javascript:void(0)) | [+](javascript:void(0)) | - | - | - | - | [+](javascript:void(0)) | [+](javascript:void(0)) | - | [+](javascript:void(0)) | - | - |
| **Toxin (18 Items)** | | | | | | | | | | | | | |
| Colibactin | *clbA* | - | - | - | - | - | - | - | - | - | - | - | - |
| Colibactin | *clbB* | - | - | - | - | - | - | - | - | - | - | - | - |
| [Colibactin](javascript:void(0)) | *clbC* | - | - | - | - | - | - | - | - | - | - | - | - |
| [Colibactin](javascript:void(0)) | *clbD* | - | - | - | - | - | - | - | - | - | - | - | - |
| [Colibactin](javascript:void(0)) | *clbE* | - | - | - | - | - | - | - | - | - | - | - | - |
| [Colibactin](javascript:void(0)) | *clbF* | - | - | - | - | - | - | - | - | - | - | - | - |
| [Colibactin](javascript:void(0)) | *clbG* | - | - | - | - | - | - | - | - | - | - | - | - |
| [Colibactin](javascript:void(0)) | *clbH* | - | - | - | - | - | - | - | - | - | - | - | - |
| [Colibactin](javascript:void(0)) | *clbI* | - | - | - | - | - | - | - | - | - | - | - | - |
| [Colibactin](javascript:void(0)) | *clbJ* | - | - | - | - | - | - | - | - | - | - | - | - |
| [Colibactin](javascript:void(0)) | *clbK* | - | - | - | - | - | - | - | - | - | - | - | - |
| [Colibactin](javascript:void(0)) | *clbL* | - | - | - | - | - | - | - | - | - | - | - | - |
| [Colibactin](javascript:void(0)) | *clbM* | - | - | - | - | - | - | - | - | - | - | - | - |
| [Colibactin](javascript:void(0)) | *clbN* | - | - | - | - | - | - | - | - | - | - | - | - |
| [Colibactin](javascript:void(0)) | *clbO* | - | - | - | - | - | - | - | - | - | - | - | - |
| [Colibactin](javascript:void(0)) | *clbP* | - | - | - | - | - | - | - | - | - | - | - | - |
| [Colibactin](javascript:void(0)) | *clbQ* | - | - | - | - | - | - | - | - | - | - | - | - |
| [Colibactin](javascript:void(0)) | *clbS* | - |  | - |  | - | - |  | - | - | - | - | - |
| **Enzyme (1 Item)** | | | | | | | | | | | | | |
| [Streptococcal enolase](javascript:void(0)) | *eno* | [+](javascript:void(0)) | - | - | - | - | - | - | - | - | - | - | - |
| Fimbrial adherence determinants (2 Items) | | | | | | | | | | | | | |
| Stc [(Salmonella)](javascript:void(0)) | *stcB* | [+](javascript:void(0)) |  | - | - | - | [+](javascript:void(0)) |  | [+](javascript:void(0)) |  | - | - | - |
|  |  |  |  |  |  |  |  |  |  |  |  |  |  |
| [Stc](javascript:void(0)) (Salmonella) | *stcC* | [+](javascript:void(0)) |  | - | - | - | [+](javascript:void(0)) | - | [+](javascript:void(0)) | - | - | - | - |
|  |  |  |  |  |  |  |  |  |  |  |  |  |  |
